# Drivers and Decoupling Effects of PM_2.5_ Emissions in China: An Application of the Generalized Divisia Index

**DOI:** 10.3390/ijerph20020921

**Published:** 2023-01-04

**Authors:** Shangjiu Wang, Shaohua Zhang, Liang Cheng

**Affiliations:** 1 School of Economics and Statistics, Guangzhou University, Guangzhou 510006, China; 2 School of Mathematics and Statistics, Shaoguan University, Shaoguan 512005, China; 3 School of Political Science and Law, Shaoguan University, Shaoguan 512005, China

**Keywords:** PM_2.5_ emissions, GDIM, decoupling effect, innovation input

## Abstract

Although economic growth brings abundant material wealth, it is also associated with serious PM_2.5_ pollution. Decoupling PM_2.5_ emissions from economic development is important for China’s long-term sustainable development. In this paper, the generalized Divisia index method (GDIM) is extended by introducing innovation indicators to investigate the main drivers of PM_2.5_ pollution in China and its four subregions from 2008 to 2017. Afterwards, a GDIM-based decoupling index is developed to examine the decoupling states between PM_2.5_ emissions and economic growth and to identify the main factors leading to decoupling. The obtained results show that: (1) Innovation input scale and GDP are the main drivers for increases in PM_2.5_ emissions, while innovation input PM_2.5_ intensity, emission intensity, and emission coefficient are the main reasons for reductions in PM_2.5_ pollution. (2) China and its four subregions show general upward trends in the decoupling index, and their decoupling states turn from weak decoupling to strong decoupling. (3) Innovation input PM_2.5_ intensity, emission intensity, and emission coefficient contribute largely to the decoupling of PM_2.5_ emissions. Overall, this paper provides valuable information for mitigating haze pollution.

## 1. Introduction

Over recent decades, China has seen enormous economic growth, but also suffered from frequent and widespread haze pollution [1,2]. Fine particulate matter, i.e., PM_2.5_ (≤ 2.5 μm in aerodynamic diameter), is the primary cause of haze episodes in China [3,4]. Despite being small in size, PM_2.5_ has a strong capacity to absorb various toxic substances and may induce a variety of diseases [5,6,7]. In China, the estimated number of PM_2.5_-related deaths reached 1.1 million in 2015, increasing by 0.15 million compared with 1990 [8]. In addition to adverse effects on human health, PM_2.5_ pollution also contributes significantly to visibility degradation, climate change, and economic loss [9,10,11]. As revealed in previous research, the PM_2.5_-related economic loss in China in 2016 reached USD 101 billion, which was nearly 1% of China’s GDP [12]. Thus, PM_2.5_ pollution has threatened sustainable development in China, and how to alleviate PM_2.5_ pollution has become a vital and challenging task for the government.

Aiming to improve air quality, the Chinese government has formulated a range of policies since 2013, such as “The Air pollution prevention and control action plan” and “The Three-Year Action Plan for Winning the Battle Against Air Pollution” [13,14,15,16]. As a result of these tough policies, the annual average PM_2.5_ concentration of 339 Chinese cities dropped to 30 μg/m^3^ in 2021, a decrease of 58% (42 μg/m^3^) compared with 2013. However, the overall air quality is still poor in China; in 2021, only 1% of 339 cities met the national Grade I air quality standard (15 μg/m^3^). Particles released by the combustion of fossil fuels are the principal source of PM_2.5_ pollution; however, fossil fuels are the dominant energy upon which the Chinese economy relies [17,18,19,20,21]. To achieve the Sustainable Development Goals, China should avoid air pollution while promoting economic development. Thus, it is of paramount importance to dissociate economic growth from PM_2.5_ emissions.

The term “decoupling” implies the gradual untying of the coupled relationship between economic development and environmental impact [22]. Through comparison of variations in GDP and environmental stress metrics, the economy–environment relationship can be evaluated. Nowadays, the two most commonly used decoupling approaches are the indexes defined by the Organization for Economic Co-operation and Development (OECD) [23] and Tapio [24]. Using these two indexes, scholars can quantify the decoupling states of various environmental impacts, including ecological footprint, solid waste generation, wastewater emissions, carbon emissions, and air pollution, at different geographical scales (e.g., global, national, and regional scales) [25,26,27,28,29]. With regard to PM_2.5_ emissions, only a small number of studies have explored its decoupling effect as a result of data availability.

The decoupling approach is able to dynamically reflect the association between economic output and undesirable environmental impact, but it cannot identify the key factors that lead to changes in the relationship. For this reason, several decomposition analysis methods have been selected to detect pollution-related factors as a supplement to the decoupling method [30]. The logarithmic mean Divisia index (LMDI), which uses the log mean weight function and shows perfect performance in factor decomposition, has been shown preference in the literature [31,32]. For example, by applying the LMDI technique, Raza and Lin investigated the drivers of transport CO_2_ emissions in the case of Pakistan from 1984 to 2018 [33]; Lyu et al. identified the determinants of PM_2.5_, NOx, and SO_2_ emissions in China from 1997 to 2012 [34]; and Tian et al. measured the contributions of factors governing wastewater discharge in China at the provincial level from 2011 to 2017 [35]. However, the LMDI method is unable to reveal the effects of multiple absolute indicators [22], and contradictory results may occur when the LMDI decomposition forms are different [36]. Thus, to address these drawbacks, the generalized Divisia index method (GDIM) was presented by Vaninsky [37]. Subsequently, the application of this approach is gradually growing [37,38,39,40,41], with only a few cases focusing on PM_2.5_ pollution [28,42,43].

Moreover, though a large body of literature has explored the determinants of PM_2.5_ pollution, few studies have considered the roles of technological innovation. As technology can be classified into production and abatement technology, technological innovation plays a dual role in affecting air pollution [38,44]. On one hand, innovation in production technology can improve productivity, which facilitates production expansion and generates more pollutants. On the other hand, innovation in abatement technology can help reduce pollutant emissions directly. Therefore, the investment preference of technology largely determines its impact direction on air pollution. Taking R&D expenditure as a proxy for technological innovation, Chen et al. found that technological innovation can alleviate PM_2.5_ pollution [45]. However, Shao et al. [46] and Liu et al. [47] argued that different innovation factors related to R&D expenditure have various effects on carbon emissions [47]. The existing studies lack a comprehensive analysis of the impacts of innovation factors on PM_2.5_ emissions. It is thus critical to fill this gap for a better understanding of the reasons affecting changes in PM_2.5_ pollution levels.

The present study is concerned with the determinants and decoupling effects of PM_2.5_ emissions in China and its four subregions from 2008 to 2017. Firstly, the GDIM technique is used to determine the key factors affecting PM_2.5_ emissions and weigh their contributions, with special attention to the impacts of three innovation factors, namely, innovation input scale (i.e., total R&D expenditure), innovation input PM_2.5_ intensity (i.e., PM_2.5_ emissions per unit of R&D expenditure), and innovation input efficiency (i.e., GDP per unit of R&D expenditure). Secondly, a decoupling index was developed based on the GDIM technique to reveal the decoupling effect of PM_2.5_ emissions in different periods and regions. The possible novelties of this paper are as follows: (1) Different from previous studies that only consider the traditional drivers of PM_2.5_ emission changes such as GDP, energy con-sumption scale, emission intensity, emission coefficient, and energy intensity, three innovation factors (i.e., innovation input scale, innovation input PM_2.5_ intensity and innovation input efficiency) were introduced to the existing GDIM model. For the first time, the contributions of innovation factors to changes in PM_2.5_ emissions are comprehensively examined. (2) To disclose the regional and temporal heterogeneity of the relationship of PM_2.5_ emissions with economic growth or other factors, China was divided into four subregions and the whole period was divided into several subperiods.

The rest of the paper is arranged as follows: Section 2 describes the research methods and data sources used. Section 3 states and discusses the empirical results. Section 4 presents the conclusions and policy implications.

## 2. Methods and Data

### 2.1. GDIM Method

Index decomposition analysis (IDA) is a mature decomposition analysis method that has been widely used to identify the effects of different drivers on changes in PM_2.5_ emissions [8,28,42]. Among the specific IDA methods, the GDIM method proposed by Vaninsky [37] has the following advantages over other IDA methods. First, GDIM allows multiple absolute and relative indicators to be incorporated simultaneously into the target variable [38,48,49]. Second, GDIM can solve factor interdependence problems that occur in other IDA methods [39,50,51]. According to Vaninsky [37], the mapping relationship between the target variable Z and factor variables X can be expressed as:

(1) $$Z=f\left(X\right)=f\left(X_{1},X_{2},\cdots ,X_{n}\right)$$

(2) $$\Delta Z=Z_{t}-Z_{0}=\int_{L}dZ=\sum_{i=1}^{n}\Delta Z\left(X_{i}\right)=\sum_{i=1}^{n}\int_{L}f_{i}^{\prime }dX_{i}$$

where ΔZ is the change in the target variable from the current time to the reference time, $\Delta Z\left(X_{i}\right)$ is the contribution of $X_{i}$ to Z, and L represents the time. $f_{i}^{\prime }$ is the partial derivative of $f\left(X_{1},X_{2},\cdots ,X_{n}\right)$ with respect to $X_{i}$. Given $X_{i}=X_{i}\left(t\right)$, it follows that

(3) $$\Delta Z\left(X_{i}\right)=\int_{L}f_{i}^{\prime }dX_{i}=\int_{L}f_{i}^{\prime }X_{i}^{\prime }dt$$

Equation (2) can be expressed in vector form:

(4) $$\Delta Z=\int_{L}\nabla Z^{T}dX$$

where $\nabla Z=<f_{1}^{\prime },\cdots ,f_{n}^{\prime }>$ is a column gradient vector of $f\left(X_{1},X_{2},\cdots ,X_{n}\right)$.

As Vaninsky [37] points out, the decomposition above does not fully take interdependence into account. Therefore, Equation (5) is added to restrict the relationship between the decomposition factors.

(5) $$\Phi_{j}\left(X_{1},X_{2},\cdots ,X_{n}\right)=0,j=1,\cdots ,k$$

Equation (5) can be rewritten in vector form:

(6) Φ(X)=0

As a result, the GDIM decomposition of the target variable Z is

(7) $$\Delta Z\left[X|\Phi \right]=\int_{Z}\nabla Z^{T}\left(I-\Phi_{X}\Phi_{X}^{+}\right)dX$$

where $\Phi_{X}$ is the Jacobian matrix of Φ(X), I is the unit matrix, and $\Phi_{X}^{+}$ is the generalized inverse of $\Phi_{X}$. If $\Phi_{X}$ has full column rank, then $\Phi_{X}^{+}={\left(\Phi_{X}^{T}\Phi_{X}\right)}^{-1}\Phi_{X}^{T}$.

### 2.2. Decomposition of PM_2.5_ Emission Factors

The generalized Divisia index method (GDIM) is an effective decomposition approach that links the changes in pollutant emissions with socio-economic factors through the deformation of Kaya identity [28,42,43]. In contrast to classical econometric models, the GDIM approach mainly decomposes PM_2.5_ emissions based on time-series data into different influencing factors without residual terms. In this paper, the GDIM model is used to investigate the impacts of the following socio-economic drivers on PM_2.5_ emissions. According to the basic principles of GDIM, PM_2.5_ emissions can be decomposed into the following forms:

(8) $$PM=G\times \left(\frac{PM}{G}\right)=E\times \left(\frac{PM}{E}\right)=R\times \left(\frac{PM}{R}\right)\;=G\times PMG=E\times PME=R\times PMR$$

(9) $$EI=\frac{E}{G}=\left(\frac{PM}{G}\right)/\left(\frac{PM}{E}\right)=\frac{PMG}{PME}$$

(10) $$RE=\frac{G}{R}=\left(\frac{PM}{R}\right)/\left(\frac{PM}{G}\right)=\frac{PMR}{PMG}$$

Table 1 displays the definitions of the variables in Equations (8)–(10). Among these factors, G, E, PMG, PME, and EI have been frequently examined in previous relevant studies [8,28,52], but R, PMR, and RE have been somewhat overlooked in the existing index decomposition literature on PM_2.5_ emissions. In the GDIM model, G, E, and R are absolute quantitative factors, while PMG, PME, PMR, EI, and RE are relative quantitative factors.

Then, Equations (8)–(10) can be transformed into the following forms:

(11) PM=G×PMG

(12) G×PMG−E×PME=0

(13) G×PMG−R×PMR=0

(14) G−R×RE=0

(15) E−G×EI=0

Let the function PM(X) denote the response of indicator X to variations in PM_2.5_ emissions; then, the gradient of PM(X) and the Jacobean matrix consisting of the relevant impact indicators can be constructed using Equations (11)–(15):

(16) $$\nabla PM={\left(PMG,G,0,\;0,\;0,\;0,\;0,\;0\right)}^{T}$$

(17) $$\Phi_{X}={\left(\begin{array}{cccccccc} PMG & G & -PME & -E & 0 & 0 & 0 & 0\\ PMG & G & 0 & 0 & -PMR & -R & 0 & 0\\ 1 & 0 & 0 & 0 & -RE & 0 & -R & 0\\ -EI & 0 & 1 & 0 & 0 & 0 & 0 & -G \end{array}\right)}^{T}$$

The Jacobean matrix $\Phi_{X}$ is composed of the partial derivative of function PM(X), which can reflect the marginal impacts of different drivers on PM_2.5_ emissions.

Following the GDIM calculation method, the changes in PM_2.5_ emissions are decomposed as follows:

(18) $$\Delta PM\left[X|\Phi \right]=\int_{L}\nabla PM^{T}\left(I-\Phi_{X}\Phi_{X}^{+}\right)dX$$

where L denotes the study period, I denotes a matrix with all diagonal elements being 1, and $\Phi_{X}^{+}$ is the generalized inverse of $\Phi_{X}$. If $\Phi_{X}$ has full column rank, then $\Phi_{X}^{+}={\left(\Phi_{X}^{T}\Phi_{X}\right)}^{-1}\Phi_{X}^{T}$.

According to Equation (18), the variations in PM_2.5_ emissions over different time spans can be decomposed into the sum of eight effects: ΔG, ΔE, ΔR, ΔPMG, ΔPME, ΔPMR, ΔEI, and ΔRE. A detailed description of the eight effects is given in Table 1. It can be found that the sum of the results of the additive decomposition of the eight factors over the same period is about the same as total variations in PM_2.5_ emissions. Therefore, the degree of influence of each driver on PM_2.5_ emissions can be calculated separately. At the same time, the primary drivers influencing the change in PM_2.5_ emissions can be found.

### 2.3. Decoupling Model Based on GDIM

This paper uses the GDIM model to calculate the contribution of each driver to the changes in PM_2.5_ emissions, but it does not provide a direct measure of the dependence of PM_2.5_ emissions on economic growth. Therefore, the decoupling method is used to investigate the economic dependence of PM_2.5_ emissions. Using the methods found in [22,52,53,54,55,56], the decoupling effect between GDP and PM_2.5_ is defined as:

(19) $$\theta =\frac{\Delta PM∕PM^{t-1}}{\Delta G∕GDP^{t-1}}=\frac{\left(PM^{t}-PM^{t-1}\right)∕PM^{t-1}}{\left(GDP^{t}-GDP^{t-1}\right)∕GDP^{t-1}}$$

where, $PM^{t}$ ($GDP^{t}$) and $PM^{t-1}$ ($GDP^{t-1}$) represent the PM_2.5_ emissions (GDP) during time *t* and *t* − 1, respectively. Based on Equation (18), ΔPM can be expressed as

(20) ΔPM=ΔG+ΔE+ΔR+ΔPMG+ΔPME+ΔPMR+ΔEI+ΔRE

Excluding the impact of economic growth on the changes in PM_2.5_ emissions, Equation (18) can be written as

(21) ΔF=ΔPM−ΔG=ΔE+ΔR+ΔPMG+ΔPME+ΔPMR+ΔEI+ΔRE

Finally, the decoupling index between PM_2.5_ emissions and economic growth is defined as

(22) $$DI=-\frac{\Delta F/PM^{t-1}}{\Delta G/GDP^{t-1}}=-\frac{\left(\Delta E+\Delta R+\Delta PMG+\Delta PME+\Delta PMR+\Delta EI+\Delta RE\right)/PM^{t-1}}{\left(GDP^{t}-GDP^{t-1}\right)/GDP^{t-1}}\;=DI_{E}+DI_{R}+DI_{PMG}+DI_{PME}+DI_{PMR}+DI_{EI}+DI_{RE}$$

where DI is called the decoupling effort index. DI≤0 denotes “no decoupling”, 0<DI<1 refers to “weak decoupling”, and DI≥1 represents “strong decoupling”. ΔF means the changes in PM_2.5_ emissions due to the remaining drivers after the exclusion of GDP. $DI_{E}$, $DI_{R}$, $DI_{PMG}$, $DI_{PME}$, $DI_{PMR}$, $DI_{EI},$ and $DI_{RE}$ indicate the contributions of energy consumption scale, innovation input scale, emission intensity, emission coefficient, innovation input PM_2.5_ intensity, energy intensity, and innovation input efficiency, respectively, to the decoupling of PM_2.5_ emissions. The DI index has the following advantages: (1) Combining the strengths of the OECD and Tapio approaches, it measures the decoupling of socio-economic development and PM_2.5_ emissions. (2) The effect of GDP on changes in PM_2.5_ emissions is eliminated during the calculation of the DI index. (3) From Equation (22), the contributions of various factors (e.g., energy consumption scale) to the decoupling of PM_2.5_ emissions can be obtained.

### 2.4. Data Sources

Due to the fact that PM_2.5_ emission data are not easily obtained, 30 provincial-level regions in mainland China from 2008 to 2017 were selected as the study area for this paper (Tibet was excluded due to a lack of data). Socio-economic data, including GDP, energy consumption, and R&D expenditure, are taken from the China Statistical Yearbook (2009–2018), the China Energy Statistical Yearbook (2009–2018), and the China Science and Technology Statistical Yearbook (2009–2018). The PM_2.5_ emission data from 2008 to 2017 are obtained from the Multi-resolution Emission Inventory of China (MEIC: http://meicmodel.org.cn, accessed on 7 August 2022), a project developed by Tsinghua University [55,57,58,59]. To remove the effect of price volatility, GDP and R&D expenditure are expressed at 2008 constant prices. In particular, following the principles of classification in the literature [52], the whole of China is divided into four subregions, namely, Eastern, Central, Western and Northeastern China.

## 3. Results and Discussion

### 3.1. Decomposition Analysis

#### 3.1.1. Year-by-Year Decomposition of PM_2.5_ Emissions

**Figure 1:**
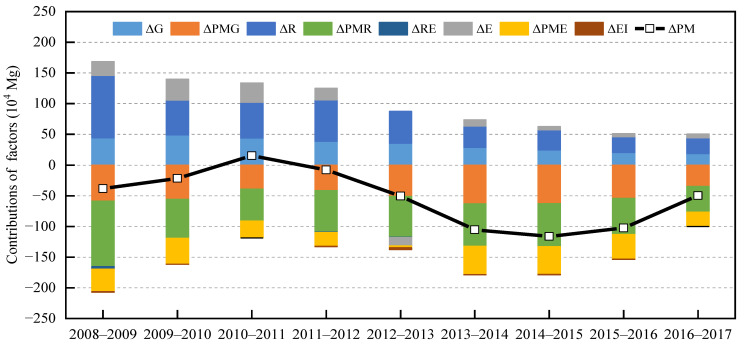
Decomposition results in China from 2008 to 2017 (Δ*G*, Δ*PMG*, Δ*R*, Δ*PMR*, Δ*RE*, Δ*E*, Δ*PME*, and Δ*EI* denote the effects of changes in GDP, emission intensity, innovation input scale, innovation input PM_2.5_ intensity, innovation input efficiency, energy consumption scale, emission coefficient, and energy intensity on the changes in PM_2.5_ emissions, respectively. Δ*PM* indicates the changes in PM_2.5_ emissions).

**Figure A1:**
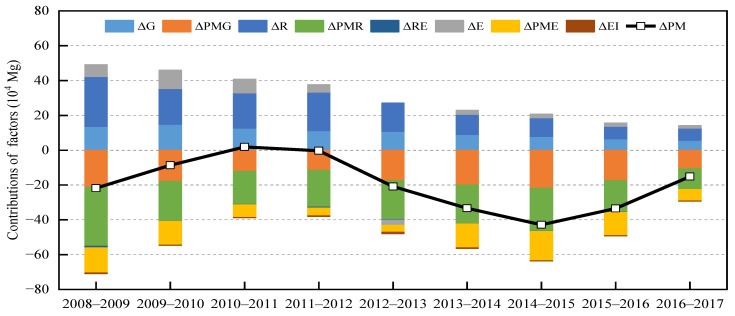
Decomposition results in Eastern China from 2008 to 2017 (Δ*G*, Δ*PMG*, Δ*R*, Δ*PMR*, Δ*RE*, Δ*E*, Δ*PME* and Δ*EI* denote the effects of changes in GDP, emission intensity, innovation input scale, innovation input PM_2.5_ intensity, innovation input efficiency, energy consumption scale, emission coefficient, and energy intensity on the changes in PM_2.5_ emissions, respectively. Δ*PM* indicates the changes in PM_2.5_ emissions).

**Figure A2:**
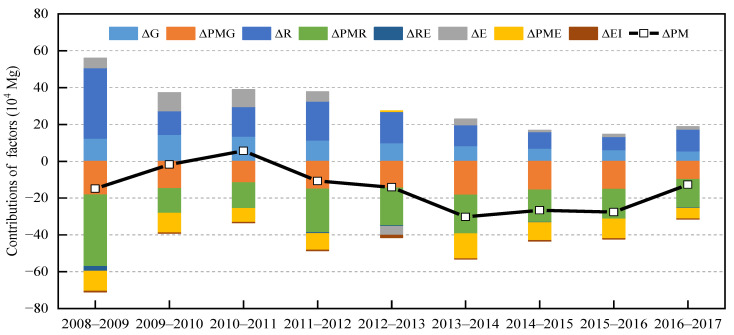
Decomposition results in Central China from 2008 to 2017 (Δ*G*, Δ*PMG*, Δ*R*, Δ*PMR*, Δ*RE*, Δ*E*, Δ*PME* and Δ*EI* denote the effects of changes in GDP, emission intensity, innovation input scale, innovation input PM_2.5_ intensity, innovation input efficiency, energy consumption scale, emission coefficient, and energy intensity on the changes in PM_2.5_ emissions, respectively. Δ*PM* indicates the changes in PM_2.5_ emissions).

**Figure A3:**
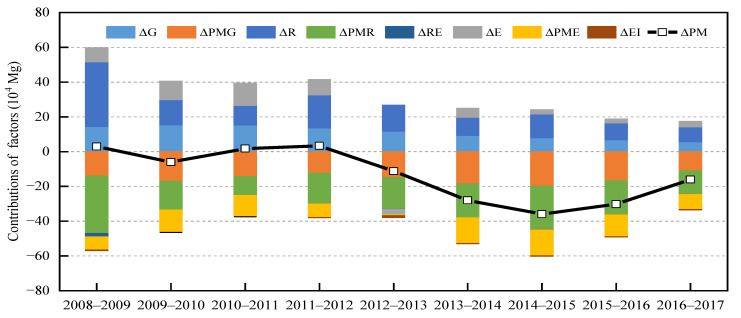
Decomposition results in Western China from 2008 to 2017 (Δ*G*, Δ*PMG*, Δ*R*, Δ*PMR*, Δ*RE*, Δ*E*, ΔP*ME* and Δ*EI* denote the effects of changes in GDP, emission intensity, innovation input scale, innovation input PM_2.5_ intensity, innovation input efficiency, energy consumption scale, emission coefficient, and energy intensity on the changes in PM_2.5_ emissions, respectively. Δ*PM* indicates the changes in PM_2.5_ emissions).

**Figure A4:**
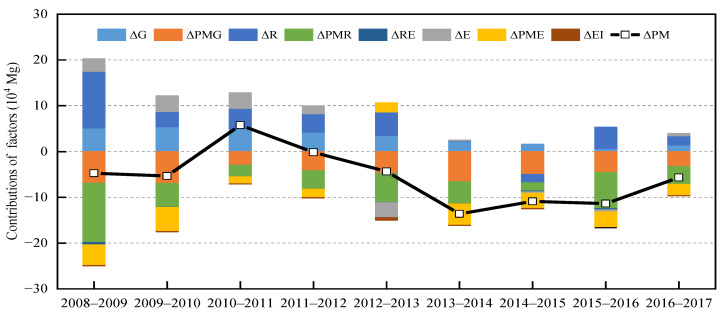
Decomposition results in Northeast China from 2008 to 2017 (Δ*G*, Δ*PMG*, Δ*R*, Δ*PMR*, Δ*RE*, Δ*E*, Δ*PME* and Δ*EI* denote the effects of changes in GDP, emission intensity, innovation input scale, innovation input PM_2.5_ intensity, innovation input efficiency, energy consumption scale, emission coefficient, and energy intensity on the changes in PM_2.5_ emissions, respectively. Δ*PM* indicates the changes in PM_2.5_ emissions).

In order to identify the main drivers influencing the variations in PM_2.5_ emissions, the contributions of eight drivers (e.g., GDP (Δ*G*), energy consumption scale (Δ*E*), and innovation input scale (Δ*R*), etc.) were calculated using the R software according to Equation (18). The year-by-year decomposition of PM_2.5_ emissions in China and its four subregions are shown in Figure 1 (Table A1) and Figure A1, Figure A2, Figure A3 and Figure A4 (Appendix A). It can be found that the effects of all the factors on PM_2.5_ emissions in the four subregions are similar to the situation in the whole of China. Consequently, this paper takes the whole country as an example for analysis.

As shown in Figure 1 (Table A1), the contribution of innovation input scale (Δ*R*) to PM_2.5_ emissions is positive for the 2008–2017 period, and its annual average contribution is 506,105.6 Mg, which shows that innovation input scale (Δ*R*) has resulted in a significant increase in PM_2.5_ emissions [60]. This is due to the fact that during the 2008–2017 period, R&D expenditure may have been spent more on promoting technological advances in production than on green technologies, stimulating the expansion of production and leading to increases in PM_2.5_ emissions [44]. Furthermore, GDP (Δ*G*) plays a crucial role in increasing PM_2.5_ emissions, and its annual average contribution is 337,518.7 Mg. In contrast, the energy consumption scale (Δ*E*) is observed to play a minor role in promoting PM_2.5_ emissions, and its annual average contribution is 134,278 Mg, except for 2012–2013.

Figure 1 (Table A1) shows that the major drivers for reductions in PM_2.5_ emissions are innovation input PM_2.5_ intensity (Δ*PMR*), emission intensity (Δ*PMG*), and emission coefficient (Δ*PME*). Innovation input PM_2.5_ intensity (Δ*PMR*) plays the most important role in reducing PM_2.5_ emissions from 2008 to 2017. It is found that from 2008 to 2009, innovation input PM_2.5_ intensity (Δ*PMR*) has led to a significant reduction in PM_2.5_ emissions equal to the amount of 1,069,357 Mg. Subsequently, the contribution of innovation input PM_2.5_ intensity (Δ*PMR*) presents a stable trend, with an average annual decrease of 638,940.9 Mg from 2009 to 2016. After 2016, the reduction in PM_2.5_ emissions attributed to innovation input PM_2.5_ intensity (Δ*PMR*) decreases slightly. With increasing R&D expenditure, the technology innovations of enterprises have been improved, in turn increasing the efficiency of factor utilization and reducing PM_2.5_ emissions [44,45]. In line with previous studies, emission intensity (Δ*PMG*) also has a negative impact on PM_2.5_ emissions [30,35,53]. Specifically, the reduced level of PM_2.5_ emissions due to emission intensity (Δ*PMG*) is 51,253,335 Mg per year on average, implying that reducing emission intensity (Δ*PMG*) can effectively improve air quality. In addition, the emission coefficient (Δ*PME*) is another significant driver of PM_2.5_ emission reduction, in line with the findings in the literature [35]. It is observed that the emission coefficient (Δ*PME*) reduced PM_2.5_ emissions for this entire term by an average of 354,372.4 Mg a year, except for 2012–2013. This is because the government of China has undertaken a tremendous amount of work on energy transformation. For instance, the government implemented the Golden Sun Demonstration Project in 2009, increasing the applications of solar energy [61].

Overall, energy intensity (Δ*EI*) has a relatively small effect on the reduction in PM_2.5_ emissions. However, it led to a relatively large reduction in PM_2.5_ emissions from 2012 to 2013, with a reduction of 32,855.3 Mg. The reason for this may be that, in 2011, the Chinese government established a national mandatory goal of reducing energy intensity (Δ*EI*) by 16% by 2015 [62,63]. Similarly, innovation input efficiency (Δ*RE*) makes a small contribution to the reduction in PM_2.5_ emissions. These findings imply that there is still much room for improvement in energy intensity (Δ*EI*) and innovation input efficiency (Δ*RE*).

#### 3.1.2. Comparison of Decomposition Results of the Changes in PM_2.5_ Emissions for China and Its Four Subregions over Different Time Periods

**Figure 2:**
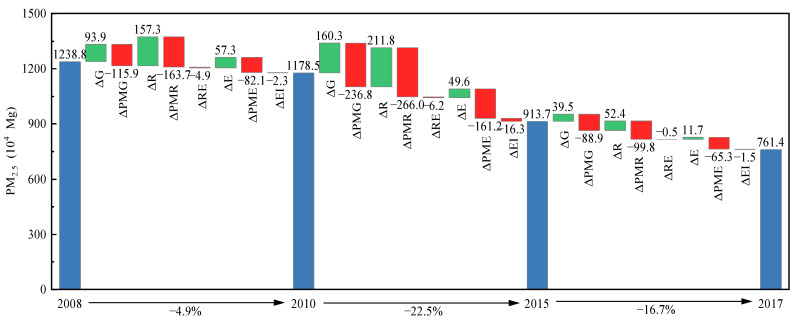
Decomposition results in China over different periods (Δ*G*, Δ*PMG*, Δ*R*, Δ*PMR*, Δ*RE*, Δ*E*, Δ*PME* and Δ*EI* denote the effects of changes in GDP, emission intensity, innovation input scale, innovation input PM_2.5_ intensity, innovation input efficiency, energy consumption scale, emission coefficient, and energy intensity on the changes in PM_2.5_ emissions, respectively).

**Figure 3:**
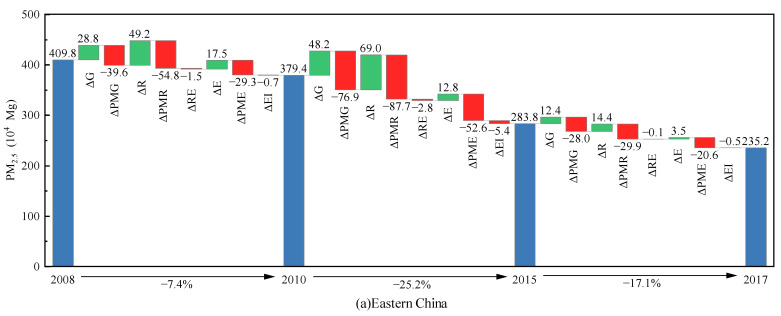

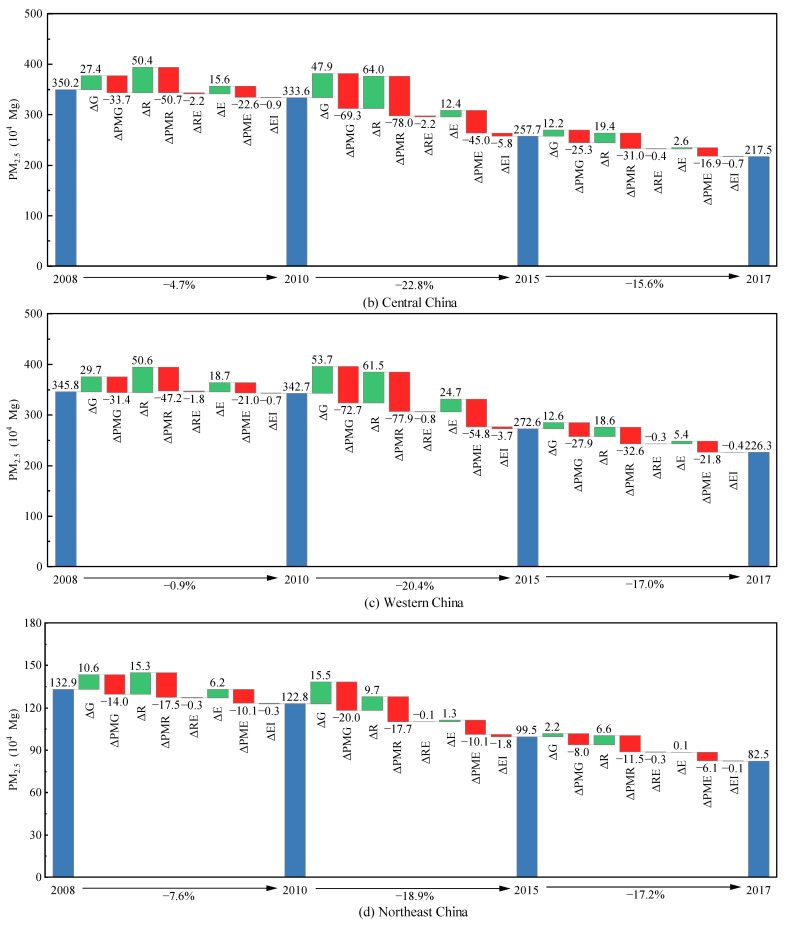
Decomposition results of the four subregions over different periods (Δ*G*, Δ*PMG*, Δ*R*, Δ*PMR*, Δ*RE*, Δ*E*, Δ*PME* and Δ*EI* denote the effects of changes in GDP, emission intensity, innovation input scale, innovation input PM_2.5_ intensity, innovation input efficiency, energy consumption scale, emission coefficient, and energy intensity on the changes in PM_2.5_ emissions, respectively).

China’s “Five-Year Plan” proposes medium targets for socio-economic development. In line with the “Five-Year Plan”, the study period (i.e., 2008–2017) is divided into three subperiods: 2008–2010, 2010–2015, and 2015–2017. Figure 2 (Table A2) and Figure 3 (Table A3) illustrate the impacts of different factors on the variations in PM_2.5_ emissions in China and its four subregions (Eastern, Central, Western and Northeast China) across three subperiods.

As shown in Figure 2 (Table A2), China’s PM_2.5_ emissions continued to decline over the whole period, from 12,387,984 Mg in 2008 to 76,138,818 Mg in 2017. The reduction rates of China’s PM_2.5_ emissions over different periods are 2.5% (2008–2010), 5.0% (2010–2015) and 8.7% (2015–2017) per year on average, respectively. Three absolute quantities, i.e., innovation input scale (Δ*R*), GDP (Δ*G*), and energy consumption scale (Δ*E*), are the driving factors that lead to rises in PM_2.5_ emissions. In particular, comparing the decomposition results for 2008–2010 and 2015–2017, it can be seen that the impacts of these three absolute quantities on the variations in PM_2.5_ emissions become smaller from 2015 to 2017. Innovation input PM_2.5_ intensity (Δ*PMR*) is the largest driver of China’s PM_2.5_ emissions reduction. The reductions in PM_2.5_ emissions due to innovation input PM_2.5_ intensity (Δ*PMR*) are 1637,000 Mg, 266,000 Mg and 998,000 Mg for 2008–2010, 2010–2015 and 2015–2017, respectively. Emission intensity (Δ*PMG*) and emission coefficient (Δ*PME*) are the other two dominant factors curbing PM_2.5_ emissions. Since the 12th Five-Year Plan, China’s government has implemented a wide range of policies to curb pollution, for example, setting strict PM_2.5_ emission reduction targets, optimizing energy structure, and promoting the clean utilization of coal [22], which helps to decrease PM_2.5_ emissions [64]. In contrast, the influences of energy intensity (Δ*EI*) and innovation input efficiency (Δ*RE*) are relatively small.

Considering the regional heterogeneity of factors impacting PM_2.5_ emissions, this paper further breaks down the PM_2.5_ emissions of the four subregions over three subperiods (Figure 3). It can be observed that PM_2.5_ emissions in the four subregions show similar changing trends. Specifically, since the 12th Five-Year Plan, PM_2.5_ emissions in all four subregions have shown obvious downward trends, with similar decreasing rates. Furthermore, the main factors increasing or decreasing PM_2.5_ pollution are also similar in the four subregions, although regional variations are found regarding the magnitude of each factor driving PM_2.5_ emission changes.

As illustrated in Figure 3 (Table A3), the innovation input scale (Δ*R*) provides the largest positive contribution to PM_2.5_ pollution. In particular, over the 2010–2015 period, the innovation input scale (Δ*R*) leads to an increase in PM_2.5_ pollution of 69.0, 64.0, and 61.5 Mg for Eastern China, Central China, and Northeast China, respectively. GDP (Δ*G*) is also a key driver leading to a rise in PM_2.5_ emissions. In particular, the rise in PM_2.5_ pollution due to growth of GDP (Δ*G*) in Western China reached 53.7 Mg during the 2010–2015 period. In addition, the energy consumption scale (Δ*E*) also promotes an increase in PM_2.5_ pollution. Among the four subregions, Western China has the largest PM_2.5_ emissions due to its high energy consumption (Δ*E*). Especially during the 2010–2015 and 2015–2017 periods, PM_2.5_ emissions due to energy consumption scale (Δ*E*) are 18 times and 50 times higher in Western China than in Northeast China, respectively. This is because China has accelerated the development of the western region since the 18th CPC National Congress [65], leading to a considerable number of energy-intensive enterprises shifting from eastern regions to western regions [66]. Innovation input PM_2.5_ intensity (Δ*PMR*), emission intensity (Δ*PMG*), and emission coefficient (Δ*PME*) are the primary drivers of PM_2.5_ pollution reduction for the four subregions, with innovation input PM_2.5_ intensity (Δ*PMR*) having the strongest inhibitory effect, followed by emission intensity (Δ*PMG*) and emission coefficient (Δ*PME*). In detail, PM_2.5_ emission reductions due to innovation input PM_2.5_ intensity (Δ*PMR*), emission intensity (Δ*PMG*) and emission coefficient (Δ*PME*) are significantly lower in Northeast China than in the other three economic regions throughout the period. This is because economic development in Northeast China relies heavily on heavy and energy-intensive industries, and PM_2.5_ pollution is more serious in this region [67]. Innovation input efficiency (Δ*RE*) and energy intensity (Δ*EI*) both have little influence on reducing PM_2.5_ pollution in all subregions. During the 2010–2015 period, the highest PM_2.5_ emission reductions caused by innovation input efficiency (Δ*RE*) and energy intensity (Δ*EI*) occur in Eastern China (2.8 Mg) and Central China (5.8 Mg), respectively.

### 3.2. Decoupling Effect Analysis

#### 3.2.1. Evaluation of Decoupling States

**Figure 4:**
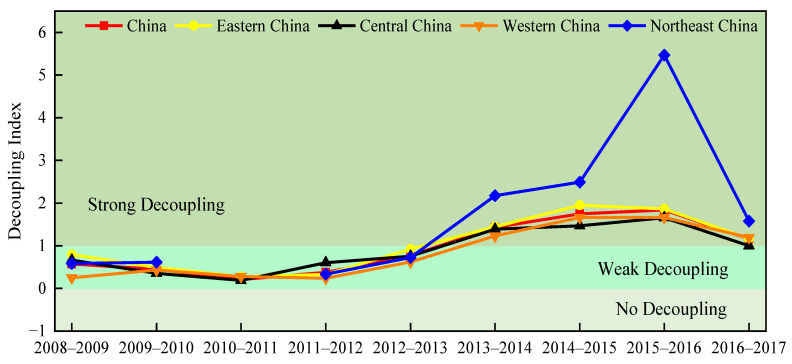
Decoupling trends of PM_2.5_ emissions from 2008 to 2017.

Figure 4 depicts the decoupling trends of PM_2.5_ emissions in China and its four subregions from 2008 to 2017. The decoupling indexes first decrease, then rise and finally decrease again, but the overall trends are upward. Specifically, from 2008 to 2009, the decoupling indexes in China and its subregions are positive and less than 1, showing a weak decoupling state, which indicates that PM_2.5_ emissions grow slowly with economic growth. From 2009 to 2011, all the decoupling indexes display obvious and continuous decline, implying the gradually weakening decoupling state in China and its four subregions. Especially in Northeast China, the decoupling index decreases from 0.62 (2009–2010) to −0.05 (2010–2011), reflecting the decoupling state changes from weak decoupling to no decoupling. The result suggests that efforts to reduce PM_2.5_ emissions in Northeast China over this period cannot curb the intensification of PM_2.5_ pollution due to economic growth [22]. From 2011 to 2015, across China and its four subregions, the decoupling indexes gradually increase and the decoupling states change from weak decoupling to strong decoupling. This can be explained by the fact that during the 2011–2015 period, the Chinese government made a great effort to reduce emissions [22,64], mitigating PM_2.5_ pollution effectively. From 2015 to 2016, all the decoupling indexes are greater than 1, indicating that China and its four subregions achieved strong decoupling. In particular, the decoupling index of Northeast China reaches the peak (5.47) during this period, which means that PM_2.5_ emissions were increasing at a much slower rate than economic growth. From 2016 to 2017, although the decoupling indexes show downward trends again, China and its four subregions still present strong decoupling.

**Figure 5:**
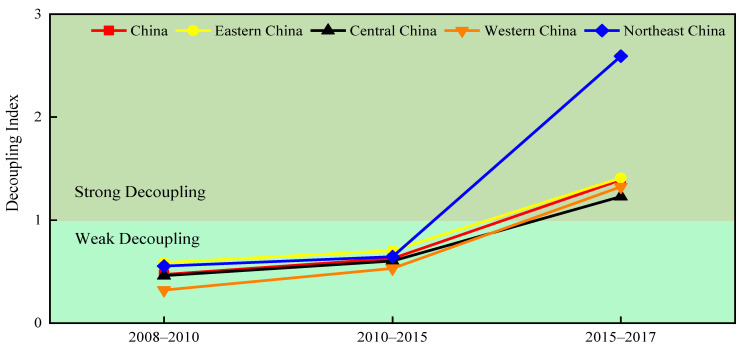
Decoupling trends of PM_2.5_ emissions in the medium term.

Figure 5 demonstrates the decoupling of PM_2.5_ emissions and economic development in the medium term. It is obvious that the decoupling states of China and its four subregions gradually improve during the 2008–2017 period. From 2008 to 2010 and 2010 to 2015, China and its subregions show weak decoupling, while from 2015 to 2017, they exhibit strong decoupling. This finding reflects the effective decoupling efforts made by China and its four subregions, so that the rise in PM_2.5_ pollution caused by economic development can be curbed. Overall, the decoupling process revealed by Figure 5 is consistent with the short-term decoupling results.

#### 3.2.2. The role of Different Drivers in Decoupling

Decoupling emphasizes the stable and sustained separation of economic development and air pollution in the relatively long term, but not the short term. For the purpose of identifying the key drivers influencing the decoupling of PM_2.5_ emissions, this paper develops a decoupling effort model based on the GDIM method. Using Equation (22), the contribution of each factor to the decoupling index is calculated. Detailed results can be found in Table 2 and Table 3.

As presented in Table 2, from 2008 to 2017, the contributions of innovation input PM_2.5_ intensity (Δ*PMR*), emission intensity (Δ*PMG*) and emission coefficient (Δ*PME*) are positive and large, implying their prominent roles in promoting the decoupling process. Meanwhile, a remarkable increase can be observed in the decoupling index across China for the 2008–2017 period, from 0.47 (2008–2010) to 1.39 (2015–2017). This may be related to the increasing contributions of the aforementioned three factors to decoupling index. During the 2015–2017 period, the contribution values of innovation input PM_2.5_ intensity (Δ*PMR*), emission intensity (Δ*PMG*), and emission coefficient (Δ*PME*) reached 0.64, 0.72, and 0.47, respectively, which are much higher rates than in the previous periods. In contrast, the innovation input scale (Δ*R*) and energy consumption scale (Δ*E*) have adverse impacts on decoupling for the whole period. During the past few years, R&D funds may have been used primarily to improve production technology [44], stimulating the expansion of production scale and thereby hindering the decoupling process in China [22].

Table 3 illustrates the roles of multiple drivers for the decoupling of PM_2.5_ emissions from economic growth in different regions over the 2008–2017 period. The four subregions present similar decoupling processes, transforming from weak decoupling to strong decoupling. It is obvious that innovation input PM_2.5_ intensity (Δ*PMR*) is the leading factor affecting the decoupling states of the four subregions in the three subperiods, except for in Northeast China from 2010 to 2015. In addition, emission intensity (Δ*PMG*) and the emission coefficient (Δ*PME*) are also dominant drivers for the decoupling of PM_2.5_ emissions in different regions over this period. Accordingly, more efforts should be focused on technological innovation, adjusting industrial structure, and promoting clean energy.

## 4. Conclusions and Policy Implications

### 4.1. Conclusions

The present study investigates the main drivers of PM_2.5_ emissions in China and its four subregions from 2008 to 2017. Then, the decoupling states between PM_2.5_ emissions and economic growth are examined and compared for China and its four subregions. Finally, the contributions of different factors to the decoupling index are quantified.

(1) Innovation input scale (Δ*R*) and GDP (Δ*G*) are the main factors for the increase in PM_2.5_ emissions. In contrast, innovation input PM_2.5_ intensity (Δ*PMR*) contributes most for the reduction in PM_2.5_ emissions, followed by emission intensity (Δ*PMG*) and emission coefficient (Δ*PME*).
(2) In the four subregions, PM_2.5_ emissions show similar changing trends, with obvious downward trends with similar rates since the implementation of 12th Five-Year Plan. In addition, the major factors increasing or mitigating PM_2.5_ pollution are also similar in the four subregions, though the magnitudes of increases or decreases shows regional variations.
(3) From 2008 to 2017, the decoupling indexes for China and its four subregions first decrease, then rise, and finally decrease again, showing overall upward trends, and the decoupling states turn from weak decoupling to strong decoupling.
(4) During the whole period, the contributions of innovation input PM_2.5_ intensity (Δ*PMR*), emission intensity (Δ*PMG*) and emission coefficient (Δ*PME*) to the decoupling are positive and large, implying their prominent roles in promoting the decoupling process.
(5) This paper has a few limitations. Firstly, due to the limitation in data availability, the effects of different types of technological innovation (e.g., production technology innovation and abatement technology innovation) on PM_2.5_ emissions are not examined precisely. To obtain more accurate results, the total R&D funds should be divided into funds for production technology and abatement technology when data is available. Secondly, factors that are not easily measured, e.g., environmental regulation, are not incorporated into the GDIM model. Considering that a variety of policies and strategies have been implemented for controlling air pollution in China, quantifying the impacts of these policies and strategies on PM_2.5_ emissions is our next concern. Lastly, the study only concentrates on China and its four subregions, without special consideration for heavily polluted areas, e.g., the Fenwei Plain. The investigation of heavily polluted areas should be considered in future work.

### 4.2. Policy Implication

Based on the above empirical results, several policy implications can be drawn as follows:

(1) The results revealed in this study suggest that the innovation input PM_2.5_ intensity (Δ*PMR*) can mitigate PM_2.5_ emissions and promote the decoupling of PM_2.5_ emissions and economic growth. Therefore, the government should provide sufficient financial and tax support, such as raising R&D expenditure on energy saving and emission reduction and encouraging enterprises to increase R&D investment in green technology innovation, so as to reduce PM_2.5_ emissions.
(2) As emission intensity (Δ*PMG*) plays a significant role in reducing PM_2.5_ emissions, it is necessary to reduce the use of traditional energy by adjusting and optimizing the industrial structure. On one hand, the government should develop high-technology industries which have low energy consumption and vigorous high-value addition. On the other hand, energy-intensive industries with high air pollutant emission intensity and backward technology should be gradually eliminated.
(3) More attention should be paid to improvements in energy structure. This is because the emission coefficient (Δ*PME*) has a significant impact on reducing PM_2.5_ pollution. Thus, promoting the utilization of cleaner and renewable energies (e.g., wind and solar energy) is an effective way to mitigate PM_2.5_ pollution.

## Figures and Tables

**Table 1:** Definition of variables in GDIM model.

Variable	Definition	Effect
PM	PM2.5 emissions	Not applicable
G	Gross domestic product	Output scale effect (Δ*G*)
E	Energy consumption scale	Energy use effect (Δ*E*)
R	Innovation input scale: total R&D expenditure	Innovation input scale effect (Δ*R*)
PMG	Emission intensity: PM2.5 emissions per unit of GDP	Emission intensity effect (Δ*PMG*)
PME	Emission coefficient: PM2.5 emissions per unit of energy consumption	Emission coefficient effect (Δ*PME*)
PMR	Innovation input PM2.5 intensity: PM2.5 emissions per unit of R&D expenditure	Innovation input PM2.5 intensity effect (Δ*PMR*)
EI	Energy intensity: energy consumption per unit of GDP	Energy intensity effect (Δ*EI*)
RE	Innovation input efficiency: GDP per unit of R&D expenditure	Innovation input efficiency effect (Δ*RE*)

**Table 2:** The roles of different drivers in decoupling in China from 2008 to 2017.

Year	*DI_PMG_*	*DI_R_*	*DI_PMR_*	*DI_RE_*	*DI_E_*	*DI_PME_*	*DI_EI_*	*DI*	*DI* State
2008–2010	0.36	−0.48	0.50	0.01	−0.18	0.25	0.01	0.47	Weak Decoupling
2010–2015	0.35	−0.31	0.39	0.01	−0.07	0.24	0.02	0.63	Weak Decoupling
2015–2017	0.64	−0.38	0.72	0.00	−0.09	0.47	0.01	1.39	Strong Decoupling

Note: *DI_PMG_*, *DI_R_*, *DI_PMR_*, *DI_RE_*, *DI_E_*, *DI_PME_* and *DI_EI_* indicate the contributions of emission intensity, innovation input scale, innovation input PM_2.5_ intensity, innovation input efficiency, energy consumption scale, emission coefficient, and energy intensity to the decoupling of the PM_2.5_ emissions, respectively. *DI* denotes the decoupling index between PM_2.5_ emissions and economic growth.

**Table 3:** The roles of different drivers in decoupling across China’s four subregions from 2008 to 2017.

Regions	Year	*DI_PMG_*	*DI_R_*	*DI_PMR_*	*DI_RE_*	*DI_E_*	*DI_PME_*	*DI_EI_*	*DI*	*DI* State
Eastern China	2008–2010	0.39	−0.49	0.54	0.02	−0.17	0.29	0.01	0.59	Weak Decoupling
	2010–2015	0.38	−0.34	0.43	0.01	−0.06	0.26	0.03	0.70	Weak Decoupling
	2015–2017	0.65	−0.33	0.69	0.00	−0.08	0.47	0.01	1.41	Strong Decoupling
Central China	2008–2010	0.35	−0.53	0.53	0.02	−0.16	0.24	0.01	0.46	Weak Decoupling
	2010–2015	0.34	−0.31	0.38	0.01	−0.06	0.22	0.03	0.60	Weak Decoupling
	2015–2017	0.59	−0.46	0.73	0.01	−0.06	0.40	0.02	1.23	Strong Decoupling
Western China	2008–2010	0.31	−0.49	0.46	0.02	−0.18	0.21	0.01	0.32	Weak Decoupling
	2010–2015	0.31	−0.26	0.33	0.00	−0.11	0.24	0.02	0.53	Weak Decoupling
	2015–2017	0.63	−0.42	0.73	0.01	−0.12	0.49	0.01	1.32	Strong Decoupling
Northeast China	2008–2010	0.37	−0.41	0.47	0.01	−0.16	0.27	0.01	0.55	Weak Decoupling
	2010–2015	0.33	−0.16	0.29	0.00	−0.02	0.17	0.03	0.64	Weak Decoupling
	2015–2017	1.08	−0.89	1.54	0.04	−0.02	0.82	0.01	2.59	Strong Decoupling

Note: *DI_PMG_*, *DI_R_*, *DI_PMR_*, *DI_RE_*, *DI_E_*, *DI_PME_* and *DI_EI_* indicate the contributions of emission intensity, innovation input scale, innovation input PM_2.5_ intensity, innovation input efficiency, energy consumption scale, emission coefficient, and energy intensity to the decoupling of the PM_2.5_ emissions, respectively. *DI* denotes the decoupling index between PM_2.5_ emissions and economic growth.

## Data Availability

The data are not publicly available due to privacy restrictions.

## Appendix A

**Table A1:** Decomposition results in China from 2008 to 2017 (unit: 10^4^ Mg).

Year	Δ*PM*	Δ*G*	Δ*PMG*	Δ*R*	Δ*PMR*	Δ*RE*	Δ*E*	Δ*PME*	Δ*EI*
2008–2009	−38.4853	44.4433	−58.6860	101.7323	−106.9357	−3.8417	22.6224	−36.8659	−0.9540
2009–2010	−21.8165	49.0523	−55.9686	56.8871	−63.2427	−0.1148	34.0345	−42.1490	−0.3152
2010–2011	15.0418	44.0476	−39.0888	58.1310	−51.9461	−0.2932	31.7324	−27.2970	−0.2442
2011–2012	−7.8906	38.7212	−41.6348	67.4470	−67.2854	−1.1331	18.9434	−22.2824	−0.6666
2012–2013	−50.4828	35.4518	−50.9645	52.0284	−66.5428	−0.6084	−13.5916	−2.9702	−3.2855
2013–2014	−105.2089	28.7909	−63.2388	34.8671	−69.0116	−0.0951	10.2452	−46.2425	−0.5240
2014–2015	−116.2647	24.4801	−62.6603	32.8300	−70.3187	−0.1759	5.3115	−45.0877	−0.6438
2015–2016	−102.7082	20.4251	−54.1442	25.4775	−58.9116	−0.0823	5.3146	−40.3454	−0.4419
2016–2017	−49.6058	18.3546	−34.7156	26.0948	−41.8572	−0.1607	6.2377	−23.2280	−0.3314

Note: Δ*G*, Δ*PMG*, Δ*R*, Δ*PMR*, Δ*RE*, Δ*E*, Δ*PME* and Δ*EI* denote the effects of changes in GDP, emission intensity, innovation input scale, innovation input PM_2.5_ intensity, innovation input efficiency, energy consumption scale, emission coefficient, and energy intensity on the changes in PM_2.5_ emissions, respectively. Δ*PM* indicates the changes in PM_2.5_ emissions.

**Table A2:** Decomposition results in China over different periods (unit: %).

Year	Δ*G*	Δ*PMG*	Δ*R*	Δ*PMR*	Δ*RE*	Δ*E*	Δ*PME*	Δ*EI*
2008–2010	7.6%	−9.4%	12.7%	−13.2%	−0.4%	4.6%	−6.6%	−0.2%
2010–2015	13.6%	−20.1%	18.0%	−22.6%	−0.5%	4.2%	−13.7%	−1.4%
2015–2017	4.3%	−9.7%	5.7%	−10.9%	−0.1%	1.3%	−7.1%	−0.2%

Note: Δ*G*, Δ*PMG*, Δ*R*, Δ*PMR*, Δ*RE*, Δ*E*, Δ*PME* and Δ*EI* denote the effects of changes in GDP, emission intensity, innovation input scale, innovation input PM_2.5_ intensity, innovation input efficiency, energy consumption scale, emission coefficient, and energy intensity on the changes in PM_2.5_ emissions, respectively.

**Table A3:** Decomposition results of China’s four subregions over different periods (unit: %).

Regions	Year	Δ*G*	Δ*PMG*	Δ*R*	Δ*PMR*	Δ*RE*	Δ*E*	Δ*PME*	Δ*EI*
Eastern China	2008–2010	7.0%	−9.7%	12.0%	−13.4%	−0.4%	4.3%	−7.1%	−0.2%
	2010–2015	12.7%	−20.3%	18.2%	−23.1%	−0.7%	3.4%	−13.9%	−1.4%
	2015–2017	4.4%	−9.9%	5.1%	−10.5%	0.0%	1.2%	−7.2%	−0.2%
Central China	2008–2010	7.8%	−9.6%	14.4%	−14.5%	−0.6%	4.4%	−6.5%	−0.2%
	2010–2015	14.4%	−20.8%	19.2%	−23.4%	−0.7%	3.7%	−13.5%	−1.7%
	2015–2017	4.7%	−9.8%	7.5%	−12.0%	−0.2%	1.0%	−6.6%	−0.3%
Western China	2008–2010	8.6%	−9.1%	14.6%	−13.7%	−0.5%	5.4%	−6.1%	−0.2%
	2010–2015	15.7%	−21.2%	17.9%	−22.7%	−0.2%	7.2%	−16.0%	−1.1%
	2015–2017	4.6%	−10.2%	6.8%	−12.0%	−0.1%	2.0%	−8.0%	−0.1%
Northeast China	2008–2010	8.0%	−10.5%	11.5%	−13.2%	−0.2%	4.6%	−7.6%	−0.2%
	2010–2015	12.6%	−16.3%	7.9%	−14.4%	−0.1%	1.0%	−8.2%	−1.5%
	2015–2017	2.2%	−8.0%	6.6%	−11.5%	−0.3%	0.1%	−6.1%	−0.1%

Note: Δ*G*, Δ*PMG*, Δ*R*, Δ*PMR*, Δ*RE*, Δ*E*, Δ*PME* and Δ*EI* denote the effects of changes in GDP, emission intensity, innovation input scale, innovation input PM_2.5_ intensity, innovation input efficiency, energy consumption scale, emission coefficient, and energy intensity on the changes in PM_2.5_ emissions, respectively.
